# Map syndrome (MYH Associated Polyposis) colorectal cancer, etiopathological connections

**Published:** 2011-02-25

**Authors:** A Bolocan, D Ion, RV Stoian, MB Serban

**Affiliations:** University Emergency Hospital Bucharest; Ⅲ'rd General Emergency Surgery Clinic, BucharestRomania

**Keywords:** genetics, recessive, hereditary

## Abstract

The case presented raised our scientific curiosity and it is worthy of being brought in front of the medical audience because of several reasons presented below. Presently, there are 3 hereditary syndromes that have a demonstrated etiological relationship with the colorectal cancer: Familiar Adenomatous Polyposis (FAP syndrome), HNPCC syndrome (Hereditary Nonpoliposis Colorectal Cancer) and MAP syndrome.

Discovered only in 2002, the MAP syndrome (MYH associated polyposis) is the first hereditary syndrome that has autosomal recessive transmission.

The APC gene can be mutated in several ways during the colonic oncogenesis: congenital in the FAP syndrome, somatic in sporadic colorectal cancers and secondary to the MYH gene inactivation in MAP syndrome. MAP phenotype is similar to the FAP phenotype because of the somatic mutations to the APC gene. Colonic polyposis is lower than FAP syndrome and appeared later, in the 40's and 50's. Colorectal cancers are frequent and discovered in the same moment as the colonic polyposis.

Patients are diagnosed mostly in cancer stages. Colonoscopy shows polyps disseminated around the entire colic frame. Treatment in these cases is total rectocolectomy with ileoanal anastomosis. When working in a general emergency surgery clinic, physicians are often faced with colorectal cancers in different evolutive stages, and mostly they are faced with their complications.

## Introduction

Starting from the ever more increased frequency of the colorectal cancers, present conceptions over the oncological disease with this localisation, try to differentiate the varied levels of oncogenesis, all the way to the most profound, the genetic one. And so, genetic and epidemiological studies [2] have shown the existence of sporadic colorectal cancers, up to 90–95% [2] from the total colorectal cancers, out of which 30% [2] have familiar involvement without being able to establish a certain genetic layer, and hereditary cancers, 5–10% [2], to which the hereditary transmission mechanism has been demonstrated. 

Presently, there are 3 hereditary syndromes that have a demonstrated an etiological relationship with the colorectal cancer: Familiar Adenomatous Polyposis (FAP syndrome), HNPCC syndrome (Hereditary Nonpoliposis Colorectal Cancer) and MAP syndrome.[1,2,5]

This paper tries to be a case report of a MAP syndrome defined on clinical criteria. This entity is interesting because of its extreme rarity and it has been defined and described for the first time in 2002.[2] So, we consider that a larger dispersal of the casuistic from this spectrum may lead to a better definition of this syndrome and may open new horizons for better knowledge of the genetic layer. 

## Case report

Patient aged 59, female, admitted in our clinic for descending colon cancer discovered during periodical colonoscopy revaluations.

From the patient's history:

- The first episode of anal bleeding took place at the age of 44, in the year 1995;
- At the age of 45, in 1996, the patient had a surgical intervention for bowl obstruction in another hospital. The post operatory diagnosis was superior rectal cancer. The attitude taken was left ovary resection and rectosygmoidectomy with coloanal anastomosis and post operatory radiotherapy. A rectal stenosis, due to the radiotherapy, led to a difficult post operatory evolution.
- At the age of 53, in 2004, the patient was urgently admitted in our clinic for bowl obstruction. The post operatory diagnosis was simultaneous transverse and descending colon. At that time, we chose large left hemicolectomy and left iliac anus.
- At the age of 57, in 2007, having entered the post operatory colonoscopy follow–up, we discovered numerous colonic polyps that were treated by multiple endoscopic resections. Their presence raised the suspicion of colonic polyposis. This presence imposed total colectomy, which the patient refused at that time
- In this situation, the management had to be limited to colonoscopy follow–ups every 6 months, having realised a real time survey of the succession polyp–polyposis–cancer.
- In 2010, at the age of 59 years old, the patient was convinced to undergo surgery as the polyps from the cecum strongly suggested malignancy transformation. Completion of the colectomy (total rectocolectomy) was chosen as the correct therapeutic choice.

From the personal pathological history of the patient, it is worth mentioning an acute colecistitis –colecistectomy at the age 29, bilateral renal lithiasis, hepatic steatosis and multiple acute pancreatitis.

From her family history we found out that she had two brothers, the first was diagnosed with colonic polyposis at the age 49 when total colectomy with ileo-rectal anastomosis was done (MAP syndrome?), and the other was diagnosed with colonic polyposis and Hodgkin lymphoma at the age of 62, both of them deceased. 

## Discussions

### Genetics

Discovered only in 2002, the MAP syndrome (MYH associated polyposis) is the first hereditary syndrome that has autosomal recessive transmission. It has almost complete penetration and variable expression. There is no subsequent mutation because the two alleles are mutated from birth. [2]

The MYH gene from chromosome 1 locus lp34, along with other genes, belongs to the DNA repairing system called ‘Base Excision Repair’ (BER). If this system is inactivated by bi–allelic mutations, several somatic mutations will have taken place, especially on the APC gene (Adenomatous Polyposis Coli). These somatic mutations on the APC gene explain the similar phenotype found also in the Familiar Adenomatous Polyposis syndrome (FAP). [2]

It is imperative to look for a MYH gene mutation when faced with a patient with colonic polyposis and a germinal mutation identified on APC gene. [1]

In addition, there is a different family history: the MAP syndrome is transmitted in a recessive manner –there is no vertical case history, the parents are heterozygous and so healthy. The transmitting risk of the disease is 25% for the MAP syndrome and 50% for the FAP syndrome in which the transmitting is dominant. [3].A patient with a clinical manifest MAP syndrome has a risk of transmitting the disease to its descendents of under 1%. Therefore, the prevalence of heterozygote mutation on MYH gene is around 1%. [5]

The APC gene can be mutated in several ways during the colonic oncogenesis: congenital in the FAP syndrome, somatic in sporadic colorectal cancers and secondary to the MYH gene inactivation in MAP syndrome. [2]

### Clinical and diagnostic aspects

MAP phenotype is similar to the FAP phenotype because of the somatic mutations to the APC gene. [4] Colonic polyposis is lower than FAP syndrome and appeared later, in the 40's and 50's. Colorectal cancers are frequent and discovered at the same moment as the colonic polyposis. This is partially explained by the fact that the parents are healthy and so there is no method for discovering the cancer. [2]

Extra–colonic symptoms are less frequent than in the FAP syndrome. Gastric and duodenal manifestations (duodenal and gastric polyps) are the most frequent which are discovered. No desmoids tumours have been found until this moment, related to the MAP syndrome.[2] Until now, the diagnosis has been based entirely on the complex sequencing of MYH gene on the circulatory lymphocytes DNA. [2]

### Management of patients with MAP syndrome

Patients are diagnosed mostly in cancer stages.[4] Colonoscopy shows polyps disseminated around the entire colic frame. Treatment in these cases is total rectocolectomy with ileoanal anastomosis.[2]

For patients who during a genetic screening test have been found with MYH gene mutations, surveillance must be completed by annual colonoscopy starting at the age 20 and biannual eso–gastro–duodeno endoscopy for extracolonic involvement.[2]

When polyps become too numerous, prophylactic surgery is imposed. The elective intervention is total rectocolectomy with ileoanal anastomosis. If the rectum is intact, it is possible to conserve it and realize a total colectomy with ileorectal anastomosis. Annual surveillance by rectoscopy is imposed in these cases.[5]

### Personal opinions

The MAP syndrome diagnose is often made rather late, towards the age of 50, because of the rarity and difficulty to proper predict or etiologically and pathogenically border this entity. The starting point of this diagnose is a constituted colonic adenocarcinoma, with or without concomitant colonic polyps of various dimensions. Clinical criteria impose a differentiation between the MAP syndrome and the HNPCC syndrome if the adenocarcinoma is not accompanied by colonic polyposis

Moreover, the genetic mechanism is essentially the same–the anomaly of a DNA repairing system (BER–Buzz Excision Repair in the MAP syndrome or MMR–MisMatch Repair in HNPCC syndrome). 

Finding colonic polyposis on colonoscopy, at an individual older than 40, must alienate the clinician from any suspicion of FAP syndrome, which is a more well known and frequent entity, in which malignancy is almost complete at the age of 30.

Complete sequencing of APC gene belonging to the chromosome 5 from the circulatory lymphocytes is now the consecrated genetic test for the FAP syndrome. Faced with a patient with colonic polyposis, but without APC gene mutation, we must search for the MYH gene mutation belonging to chromosome 1 from the circulatory lymphocytes.

Besides, personal and familiar history differs essentially. In MAP syndrome there is no vertical continuity because the transmission is autosomal recessive and so, the parents are heterozygote and healthy.

The risk of transmitting this disease to its descendent is less than 1%.

Working in a general emergency surgery clinic, we are often faced with colorectal cancers in different evolutive stages, and mostly we are faced with their complications.

This case raised our scientific curiosity and so we found it worthy of being brought in front of the medical audience because of the following reasons:

- Appearance of a second colonic cancer after 8 years from the first diagnosis;
- The lack of colonic polyposis at the moment of the first diagnosis and its presence after almost 11 years;
- Because of the patient rejection to the total colectomy, we were forced to watch the evolution of these polyps all the way to their malignancy.
- The number and aspect of these polyps excluded both the typical FAP syndrome aspect (‘polyp's carpet’) and the sporadic adenomatosic polyp that is a predecessor for most colorectal cancers.
- On the other hand, the age of the patient placed her between the two entities described before, the young age from FAP syndrome and the age over 50 from the colonic sporadic cancer.
- The two first–degree relatives diagnosed around the age 50 with colonic cancer preceded by colonic polyposis weighted also in the evocation of this probable diagnostic.
- As practitioners, we considered justified to raise the problem of etiological, pathogenic and even genetic context of a colorectal cancer at a middle–aged patient, when at this age the frequency of this disease is so rare.
- We have not had available data on the initial explorations of the colon (pan–colonoscopy) so that we could state the undisputed absence of the colonic polyps at that time, besides the explored colon during surgery. In the post–operatory period, this investigation was impossible due to a post–radiotherapy rectal stenosis
- Our first surgical intervention had an emergency character due to an occlusive complication, and so the colonoscopic investigation was yet delayed for another period of time.

We consider extremely important to apply the rule of the three moments in which you must have a pan–colonoscopy: in the pre–operatory period, during surgery or in the first 6 months post–operatory. 

In conclusion, diagnose is easy when you think of it. However, to think of such a syndrome you must have clues that will orientate ulterior diagnostic sequences–endoscopy, genetic testing.
